# Impact of comprehensive nursing care on postoperative psychological status and functional recovery after surgery for bone tumors

**DOI:** 10.1097/MD.0000000000046020

**Published:** 2025-12-12

**Authors:** Xin Xu, Xin Wang, Quanhui Liu, Lu Xie

**Affiliations:** a Joint Surgery Department, Nanhua University Affiliated Nanhua Hospital, Hengyang, Hunan Province, China; b Oncology Department, Nanhua University Affiliated Nanhua Hospital, Hengyang, Hunan Province, China.

**Keywords:** activities of daily living, bone tumor, comprehensive nursing care, limb-salvage surgery, multidisciplinary care, rehabilitation, Self-Rating Anxiety Scale, Self-Rating Depression Scale

## Abstract

This retrospective single-center study investigated the effects of comprehensive nursing care (CNC) on postoperative psychological status and functional recovery in patients undergoing limb-salvage surgery for extremity bone tumors. A total of 227 patients were included: 109 received routine nursing care (2020–2022) and 118 received CNC (2023–2024). Baseline demographic and clinical characteristics were comparable between groups. After the intervention, both groups showed significant improvements in lower extremity function, activities of daily living, and psychological measures (all *P* < .001). The intervention group, however, achieved greater gains: walking ability scores improved from 13.99 ± 2.91 to 20.12 ± 3.59, functional activity from 13.95 ± 2.84 to 20.23 ± 3.60, and activities of daily living from 61.63 ± 8.39 to 87.14 ± 9.57. Self-Rating Anxiety Scale scores decreased from 63.52 ± 7.43 to 49.96 ± 5.83 and Self-Rating Depression Scale from 69.87 ± 7.98 to 51.99 ± 5.85. These findings indicate that CNC is superior to routine care in enhancing early postoperative functional recovery, independence in daily activities, and psychological well-being.

## 1. Introduction

Bone tumors, encompassing both primary malignant neoplasms such as osteosarcoma and Ewing sarcoma, and metastatic bone lesions, represent a complex clinical challenge due to their aggressive biological behavior and the potential for profound functional impairment. In recent decades, advances in diagnostic imaging, neoadjuvant chemotherapy, and surgical techniques have shifted the surgical paradigm from amputation toward limb-salvage procedures, which aim to achieve oncologic control while preserving maximal limb function. Limb-salvage surgery (LSS) has been associated with comparable survival outcomes to amputation in appropriately selected patients; however, it is a technically demanding intervention that carries significant risks of postoperative complications, prolonged rehabilitation, and persistent functional limitations.^[1–3]^ While LSS confers substantial physical benefits by maintaining the anatomical integrity of the limb, it also introduces substantial psychosocial challenges. Patients often experience psychological distress, including anxiety, depression, and body image disturbance, due to the combined impact of the cancer diagnosis, the invasive nature of the procedure, and the prolonged recovery period. Postoperative rehabilitation is further complicated by reduced mobility, pain, and uncertainty regarding long-term functional prognosis, which can impair adherence to rehabilitation protocols and overall quality of life. Consequently, optimal postoperative outcomes require not only meticulous surgical technique but also integrated perioperative care strategies that address both physical and psychological domains.^[4,5]^

Comprehensive nursing care (CNC) is a patient-centered, multidisciplinary nursing model that integrates physical rehabilitation, psychological support, health education, and individualized nursing interventions throughout the perioperative continuum. Unlike conventional routine care, CNC emphasizes preoperative psychological assessment and counseling, individualized postoperative functional training programs, continuous pain management, complication prevention, and long-term follow-up. By proactively identifying and addressing patient-specific needs, CNC aims to improve treatment adherence, enhance psychological resilience, and accelerate functional recovery.^[6,7]^ Previous studies in orthopedic oncology and other surgical disciplines have suggested that targeted nursing interventions can improve patient-reported outcomes, reduce anxiety and depression scores, and facilitate earlier restoration of mobility. However, most available literature has focused on general orthopedic populations or amputation cases, with limited high-quality evidence evaluating the specific impact of CNC on patients undergoing LSS for bone tumors.^[8,9]^ Given the unique physical and emotional rehabilitation demands in this population, it is critical to determine whether CNC provides measurable benefits over standard postoperative care in terms of psychological status and functional recovery.

Therefore, this study aims to investigate the effect of CNC on postoperative psychological well-being and functional outcomes in patients undergoing LSS for bone tumors. By elucidating the role of CNC in optimizing recovery after LSS, the findings of this study may inform evidence-based nursing protocols and contribute to multidisciplinary strategies for improving the holistic outcomes of bone tumor patients.

## 2. Methods

### 2.1. Study design

This research was approved by the Ethics Committee of Nanhu Hospital Affiliated to Nanhu University (Approval No. NHU-EC-2020-045). This retrospective, single-center study was conducted to evaluate the impact of CNC on postoperative psychological status and functional recovery in patients with bone tumors undergoing LSS. Patients treated at our institution between January 2020 and December 2024 were included. Comprehensive nursing care was introduced in January 2023; therefore, patients admitted between January 2020 and December 2022, who received routine postoperative nursing care, were assigned to the control group (n = 109), while those admitted between January 2023 and December 2024, who received CNC, were assigned to the intervention group (n = 118).

Since this is a retrospective study, the sample size was determined based on the number of eligible patients who received treatment at our institution during the study period, rather than through prior efficacy calculations. A total of 227 patients met the inclusion and exclusion criteria, and this sample size was comparable to or larger than those reported in similar studies on limb-preserving surgery for bone tumors.^[1–3,6,9]^ The post hoc efficacy analysis based on the main outcomes (functional recovery and psychological scores) indicated that the study had sufficient statistical power (>80%) to detect clinically significant differences between groups.

Eligible patients met the following inclusion criteria: histopathologically confirmed primary bone tumor of the extremities, diagnosed through preoperative imaging, clinical evaluation, and intraoperative findings; underwent curative-intent LSS involving tumor resection and reconstruction, including internal fixation or prosthetic implantation, with the goal of preserving limb function; aged ≥ 18 years at the time of surgery; Eastern Cooperative Oncology Group (ECOG) performance status score of 0 to 2 preoperatively; and no history of severe psychiatric disorders, with sufficient cognitive ability to complete psychological evaluations and follow rehabilitation instructions. Exclusion criteria included: extensive local invasion or metastasis precluding functional limb preservation; severe cardiopulmonary, hepatic, renal, or other systemic dysfunction likely to affect perioperative safety, postoperative rehabilitation, or study outcomes; and incomplete clinical, psychological, or functional outcome data essential for analysis. The study was conducted in accordance with the ethical principles of the Declaration of Helsinki and was approved by the institutional medical ethics committee. Written informed consent was obtained from all participants prior to enrollment.

### 2.2. Nursing interventions protocol

Patients in the control group received routine postoperative nursing care, including standard wound management, medication guidance, and general rehabilitation instructions. In contrast, the intervention (study) group received a CNC program implemented in addition to routine measures, consisting of 4 key components.

Multidisciplinary intervention team: a multidisciplinary intervention team consisting of 8 members was established, including orthopedic attending physicians, rehabilitation physicians, psychosomatic medicine specialists, and senior nursing staff. The orthopedic surgeons were responsible for surgical management and perioperative medical decisions; rehabilitation physicians designed and adjusted functional training protocols; psychosomatic specialists provided psychological screening and individualized counseling; and nursing staff coordinated patient education, daily functional training supervision, and ongoing communication. For each patient, an individualized medical and nursing record was created at admission, which specified the treatment goals, rehabilitation stages, and psychological support plan. Weekly team meetings were held to review patient progress and adjust the care plan as needed, while bedside nursing staff implemented daily interventions and reported updates, ensuring continuity and consistency of comprehensive care.

Disease-specific health education: disease-specific health education was delivered preoperatively through at least 1 individualized session and 1 group session conducted by orthopedic physicians and senior nurses, covering surgical procedures, recovery timelines, potential complications, and self-care strategies. Postoperatively, nurses provided daily bedside guidance on wound care, medication, and functional training, while rehabilitation physicians reviewed and adjusted rehabilitation protocols on a weekly basis according to wound healing and functional progress.

Functional rehabilitation progressed in a staged manner: quadriceps isometric contractions initiated upon admission; active ankle dorsiflexion and plantarflexion exercises at 4 hours post-surgery; passive knee flexion training beginning on postoperative days 1 to 2 according to patient tolerance; straight leg-raising exercises on days 3 to 7; assisted ambulation with walking aids during postoperative days 8 to 14; gradual transition from partial to full weight-bearing between weeks 4 to 6 post-surgery.

Psychological interventions: mindfulness-based cognitive therapy was employed as the core psychological intervention, given its established efficacy in alleviating anxiety, depression, compulsive behaviors, and emotional dysregulation. The mindfulness-based cognitive therapy sessions involved: sitting or lying comfortably in a quiet environment with eyes closed; natural, unforced breathing; focusing attention on the breath; observing and acknowledging emotional states such as calmness or unease without judgment; redirecting attention to the breath when distracted; and daily practice for 10 to 15 minutes. Patients with significant psychological distress received targeted counseling from psychosomatic specialists to address individual emotional needs.

Post-discharge guidance and follow-up: following discharge, a patient–clinician WeChat group was established to provide continuous health education, rehabilitation progress monitoring, and disease management. Group-based virtual sessions encouraged patients to share experiences regarding daily activities, diet, emotional status, and medication adherence. Non-responders or patients requiring additional support were contacted via telephone follow-up, ensuring a multi-tiered, multi-channel, and multimodal follow-up system.

### 2.3. Assessment scales and outcome measures

Postoperative functional recovery, activities of daily living (ADL), and psychological status were evaluated using validated, widely adopted clinical assessment instruments as follows:

Lower extremity functional recovery: lower extremity function was assessed using the Lower Extremity Functional Scale, which comprises 4 dimensions with a maximum score of 25 points for each dimension. In the pain dimension, lower scores indicate better functional recovery, whereas in the remaining dimensions, higher scores denote superior lower limb function. The Lower Extremity Functional Scale has demonstrated high reliability and sensitivity in evaluating functional changes in patients undergoing orthopedic interventions.

Activities of daily living: the Barthel Index was employed to evaluate the patients’ ability to perform ADL. This instrument assesses 10 domains, including feeding, bathing, dressing, bowel and bladder control, walking on level ground, grooming, stair climbing, and transferring between bed and chair. The total score ranges from 0 to 100, with higher scores reflecting greater independence and functional capacity in daily activities.

Negative emotional states: psychological status was measured using the Self-Rating Anxiety Scale (SAS) and the Self-Rating Depression Scale (SDS). Each scale consists of 20 items, with responses scored on a 4-point Likert scale. Higher scores correspond to more severe anxiety or depressive symptoms. These instruments are widely utilized in clinical research for the quantitative assessment of negative emotional states in surgical and oncology populations.

### 2.4. Statistical analysis

Data were analyzed using SPSS version 28.0 (IBM Corp., Armonk). Continuous variables with normal distribution were expressed as mean ± standard deviation and compared between groups using the *t* test. Categorical variables were presented as frequencies and percentages, and intergroup differences were assessed using the chi-square test. In addition, analysis of covariance (ANCOVA) was performed to adjust for baseline differences where applicable (e.g., ADL scores), with baseline values entered as covariates. A 2-sided *P*-value < .05 was considered statistically significant.

## 3. Results

### 3.1. Baseline demographic, clinical, and surgical characteristics

Baseline demographic, clinical, and surgical variables were compared between the observation and control groups to ensure comparability prior to intervention (Table 1). The mean age of patients was approximately 34 years in both groups, with a balanced sex distribution, and no significant differences were observed in BMI, smoking history, or the prevalence of comorbidities such as hypertension and diabetes mellitus (all *P* > .05). Preoperative functional status, as assessed by the ECOG performance score, was similar between groups, with the majority of patients scoring 0 to 1, indicating good baseline performance. The distribution of tumor locations (femur, tibia, or other sites) and pathological types (osteosarcoma, chondrosarcoma, Ewing sarcoma, or other malignant bone tumors) was also comparable between the 2 groups (*P* > .05). Regarding intraoperative parameters, there were no statistically significant differences in mean operative duration or intraoperative blood loss between the observation and control groups (*P* > .05).

**Table 1:** Comparison of baseline demographic, clinical, and surgical characteristics between the 2 groups.

Variable	Observation group (n = 118)	Control group (n = 109)	*t*/*χ*²	*P*-value
Age (yr)	33.98 ± 16.40	33.77 ± 16.55	0.096	.924
Sex, n (%)				
Male	67 (56.78)	62 (56.88)		
Female	51 (43.22)	47 (43.12)	0.000	1.000
BMI (kg/m²)	22.47 ± 2.80	22.34 ± 2.72	0.355	.723
Smoking history, n (%)	26 (22.03)	22 (20.18)	0.032	.858
Hypertension, n (%)	19 (16.10)	18 (16.51)	0.000	1.000
Diabetes mellitus, n (%)	15 (12.71)	14 (12.84)	0.000	1.000
ECOG score (0–2), n (%)				
0–1	97 (82.20)	89 (81.65)		
2	21 (17.80)	20 (18.35)	0.000	1.000
Tumor location, n (%)				
Femur	54 (45.76)	49 (44.95)		
Tibia	45 (38.14)	42 (38.53)		
Other	19 (16.10)	18 (16.51)	0.016	.992
Pathology type, n (%)				
Osteosarcoma	67 (56.78)	61 (55.96)		
Chondrosarcoma	23 (19.49)	21 (19.27)		
Ewing sarcoma	17 (14.41)	16 (14.68)		
Other	11 (9.32)	11 (10.09)	0.046	.997
Duration of surgery (min)	177.82 ± 34.89	179.53 ± 36.03	-0.363	.717
Intraoperative blood loss (mL)	418.96 ± 95.28	426.13 ± 98.37	-0.557	.578

BMI = body mass index, ECOG = Eastern Cooperative Oncology Group performance status.

### 3.2. Functional recovery outcomes

Functional recovery parameters, including limb pain, walking ability, functional activity, and gait change, were evaluated before and after the intervention in both groups (Table 2). At baseline, no statistically significant differences were found between the observation and control groups across all 4 functional domains (*P* > .05), indicating comparable pre-intervention functional status. Following the intervention, both groups demonstrated significant improvements in all measured parameters compared with their respective baseline values (all *P* < .001). In the control group, mean limb pain scores decreased from 20.15 ± 2.54 to 15.05 ± 2.07, walking ability scores increased from 13.95 ± 2.88 to 16.48 ± 3.24, functional activity scores rose from 13.90 ± 2.82 to 16.88 ± 3.28, and gait change scores improved from 13.68 ± 2.79 to 16.75 ± 3.26. However, the magnitude of improvement was significantly greater in the observation group compared with the control group across all functional domains (*P* < .001 for all). Specifically, the observation group showed a greater reduction in limb pain (from 20.11 ± 2.56–12.11 ± 1.91) and more pronounced gains in walking ability (from 13.99 ± 2.91–20.12 ± 3.59), functional activity (from 13.95 ± 2.84–20.23 ± 3.60), and gait change (from 13.65 ± 2.77–20.07 ± 3.57).

**Table 2:** Comparison of functional recovery scores between the 2 groups before and after intervention (x̄ ± s).

Parameter	Group	Pre-intervention	Post-intervention	*t*-value	*P*-value
Limb pain	Control group	20.15 ± 2.54	15.05 ± 2.07	16.25	<.001
	Observation group	20.11 ± 2.56	12.11 ± 1.91	27.208	<.001
	*t*-value	-0.118	-11.094		
	*P*-value	.906	<.001		
Walking ability	Control group	13.95 ± 2.88	16.48 ± 3.24	-6.093	<.001
	Observation group	13.99 ± 2.91	20.12 ± 3.59	-14.409	<.001
	*t*-value	0.104	8.029		
	*P*-value	.917	<.001		
Functional activity	Control group	13.90 ± 2.82	16.88 ± 3.28	-7.193	<.001
	Observation group	13.95 ± 2.84	20.23 ± 3.60	-14.877	<.001
	*t*-value	0.133	7.336		
	*P*-value	.894	<.001		
Gait change	Control group	13.68 ± 2.79	16.75 ± 3.26	-7.47	<.001
	Observation group	13.65 ± 2.77	20.07 ± 3.57	-15.434	<.001
	*t*-value	-0.081	7.324		
	*P*-value	.935	<.001		

### 3.3. ADL

ADL scores were assessed pre- and post-intervention to evaluate the impact of treatment on patients’ self-care ability (Table 3). Before the intervention, the observation group had a slightly lower mean ADL score compared with the control group (61.63 ± 8.39 vs 64.17 ± 7.79), and this difference was statistically significant (*P* = .019). Following the intervention, both groups exhibited marked improvements in ADL scores relative to baseline (all *P* < .001). The control group’s mean ADL score increased from 64.17 ± 7.79 to 79.13 ± 8.84, while the observation group’s score improved from 61.63 ± 8.39 to 87.14 ± 9.57. Between-group comparison at post-intervention demonstrated a significantly higher mean ADL score in the observation group compared with the control group (*P* < .001), indicating superior recovery of daily functional independence. Importantly, after adjusting for baseline ADL scores using ANCOVA, the between-group difference at post-intervention remained statistically significant (*F* = 45.372, *P* < .001), confirming that the observed advantage of CNC was not attributable to baseline imbalance.

**Table 3:** Comparison of activities of daily living (ADL) scores between the 2 groups before and after intervention (x̄ ± s).

Group	Pre-intervention	Post-intervention	*t*-value	*P*-value
Control group	64.17 ± 7.79	79.13 ± 8.84	-13.261	<.001
Observation group	61.63 ± 8.39	87.14 ± 9.57	-21.773	<.001
*t*-value	-2.358	6.419		
*P*-value	.019	<.001		
ANCOVA (adjusted for baseline ADL)	–	*F* = 45.372	–	<.001

### 3.4. Univariate analysis of factors associated with postoperative wound infections

The SAS and SDS were used to evaluate changes in patients’ negative emotional states before and after intervention (Table 4). At baseline, there were no statistically significant differences in SAS or SDS scores between the observation and control groups (*P* = .960 and *P* = .150, respectively), indicating comparable levels of anxiety and depression prior to treatment. Following the intervention, both groups demonstrated significant reductions in SAS and SDS scores relative to baseline (all *P* < .001), reflecting an overall improvement in psychological status. Specifically, the mean SAS score in the control group decreased from 63.47 ± 7.54 to 57.10 ± 6.20, while the observation group showed a larger decline from 63.52 ± 7.43 to 49.96 ± 5.83. Similarly, the mean SDS score in the control group dropped from 68.37 ± 7.65 to 57.00 ± 6.81, whereas the observation group improved from 69.87 ± 7.98 to 51.99 ± 5.85. Between-group comparisons after intervention revealed significantly lower SAS and SDS scores in the observation group compared with the control group (both *P* < .001), indicating that the intervention applied to the observation group was more effective in alleviating anxiety and depressive symptoms.

**Table 4:** Comparison of SAS and SDS scores between the 2 groups before and after intervention (x̄ ± s).

Group	SAS score (Pre)	SAS score (Post)	*t*-value	*P*-value
Control group	63.47 ± 7.54	57.10 ± 6.20	6.813	<.001
Observation group	63.52 ± 7.43	49.96 ± 5.83	15.597	<.001
*t*-value	0.050	-8.920		
*P*-value	.960	<.001		
Control group	68.37 ± 7.65	57.00 ± 6.81	11.590	<.001
Observation group	69.87 ± 7.98	51.99 ± 5.85	19.630	<.001
*t*-value	1.446	-5.923		
*P*-value	.150	<.001		

SAS = Self-Rating Anxiety Scale, SDS = Self-Rating Depression Scale.

## 4. Discussion

In this retrospective analysis of patients undergoing surgery for bone tumors, CNC was associated with superior postoperative recovery across functional and psychological domains compared with routine care. Baseline demographic, clinical, and surgical variables (including age, sex, body mass index, comorbidities, ECOG performance status, tumor site, pathology, operative time, and intraoperative blood loss) were comparable between groups, supporting internal validity of the observed between-group differences. After the intervention, both groups improved significantly from baseline in limb pain, walking ability, functional activity, and gait, but the observation group achieved consistently larger gains in all domains. ADL improved in both groups, with a significantly greater post-intervention score in the observation group, indicating better restoration of independent functioning. Psychological outcomes showed parallel patterns: both anxiety (SAS) and depression (SDS) scores declined significantly in each group, with larger reductions in the comprehensive-care cohort. Collectively, these findings suggest that a structured, multidisciplinary, and patient-centered nursing model provides incremental benefits beyond standard postoperative management for orthopedic oncology patients. Nevertheless, given the retrospective design and the multi-year study period, the potential influence of temporal factors (such as the surgical team’s growing experience and gradual improvements in perioperative management) cannot be entirely excluded, and may have contributed to the observed functional gains.

Several mechanisms plausibly explain the observed effect sizes, with CNC likely playing an important role. First, comprehensive care typically includes standardized pain assessment, multimodal analgesia, and early, graded mobilization. Effective analgesia (combined with education on activity pacing) can diminish central sensitization and fear-avoidance behaviors, enabling earlier participation in rehabilitation and more rapid gains in walking ability, functional activity, and gait. Second, pathway-based health education delivered preoperatively likely improved procedural understanding and expectation setting, reducing perioperative uncertainty and fostering adherence to exercise prescriptions. Improved adherence, in turn, translates into more repetitions of therapeutic exercise, better joint range of motion, and preserved muscle strength, thereby amplifying functional benefits.^[10,11]^ Third, the integration of structured psychological support (e.g., mindfulness-based strategies and targeted counseling for high-risk patients) may have attenuated hyperarousal and negative cognitive appraisal after major oncologic surgery. Lower anxiety and depressive symptoms are associated with improved sleep quality, reduced sympathetic activation, and more favorable pain perceptions, all of which facilitate rehabilitation engagement and objective performance. The larger improvements in SAS and SDS scores in the observation group are therefore coherent with the greater functional gains recorded in the same cohort. Fourth, the use of coordinated post-discharge follow-up (e.g., digital groups and scheduled outreach) likely sustained self-management and early troubleshooting of barriers to recovery, reinforcing therapeutic behaviors during the critical subacute period.^[12,13]^

The ADL findings merit specific comment. Although the observation group began with a slightly lower mean ADL score, it surpassed the control group after intervention by a statistically and clinically meaningful margin. This indicates that CNC not only accelerates recovery but can also overcome modest baseline deficits in independence. To further address the baseline imbalance, we conducted an ANCOVA with baseline ADL as a covariate, and the superiority of the intervention group remained statistically significant after adjustment. This strengthens the robustness of our findings. Nevertheless, the baseline ADL difference underscores the importance of considering regression to the mean and, in future work, applying covariate adjustment or mixed-effects modeling with larger, multicenter prospective cohorts to confirm long-term validity.^[14,15]^

The present results are congruent with a growing body of evidence in orthopedic and oncologic rehabilitation indicating that multimodal, protocolized nursing interventions improve patient-centered outcomes relative to usual care. Prior observational cohorts and quasi-experimental studies in limb-salvage or major joint surgery populations have reported that structured perioperative pathways (combining analgesic optimization, early mobilization, and individualized exercise) yield faster gains in ambulation distance, time to independent transfers, and discharge readiness.^[16,17]^ Our study extends these findings by demonstrating parallel improvements across several granular functional domains (pain, walking ability, functional activity, and gait) rather than single composite endpoints, strengthening the case that the benefit is broad rather than domain-specific. The psychological results align with reports that perioperative anxiety and depressive symptoms are modifiable determinants of rehabilitation performance.^[18]^ Programs embedding cognitive-behavioral elements, mindfulness practices, or systematic psychoeducation have shown reductions in anxiety and depressive symptom severity after major surgery; these changes have been linked to improved adherence with physical therapy and lower perceived pain. The larger effect sizes in our observation group for both SAS and SDS mirror these patterns and suggest that integrating psychological care into nursing practice is an efficient lever to improve functional outcomes.

Not all studies have shown uniform benefit across endpoints. Some randomized trials in general orthopedic populations have reported equivalent long-term function despite early advantages with enhanced recovery protocols, potentially due to ceiling effects once tissue healing reaches a plateau. Our results (captured in the early postoperative trajectory) are most consistent with accelerated recovery, which may or may not translate into long-term superiority. Additionally, heterogeneity in program components (e.g., intensity of supervised exercise, availability of psychosomatic consultation, digital follow-up fidelity) can influence effect sizes. Compared with such heterogeneous interventions, the comprehensive approach evaluated here appears to have ensured sufficient implementation intensity across physical and psychological domains to achieve consistent between-group differences. For clinical practice, these findings support adoption of CNC as a standard adjunct to surgical management of bone tumors. Key operational elements include: preoperative pathway-based education; multimodal analgesia and early, graded mobilization; individualized, criterion-based progression of functional training; embedded psychological support with routine screening and brief interventions; and structured post-discharge follow-up to sustain adherence. Implementing these elements through an interdisciplinary team can accelerate recovery of independence, reduce symptom burden, and improve patient-reported outcomes without altering surgical technique or resource-intensive inpatient pathways.

This study possesses several methodological strengths. Baseline demographic, clinical, and operative characteristics were well balanced between groups, minimizing confounding from patient- or procedure-related factors. Outcomes were comprehensively assessed, encompassing multiple functional domains, ADL, and validated psychological measures, thereby capturing both physical and mental health dimensions of recovery. The consistent, directionally concordant effects observed across endpoints enhance the internal coherence of the findings. Additionally, the application of standardized scales (e.g., SAS, SDS) and clearly defined pre- and post-intervention comparisons supports reproducibility and facilitates comparison with other studies. However, limitations must be acknowledged. The retrospective, single-center design increases the potential for selection bias and limits external generalizability, particularly to institutions with different rehabilitation resources or nursing models. To minimize bias, we applied strict inclusion and exclusion criteria, excluded patients with incomplete clinical or follow-up data, and ensured that all 227 eligible cases during the study period were included without loss to follow-up, thereby maintaining data integrity. Reliance on patient-reported measures may introduce response bias. Statistical analysis primarily employed repeated *t* tests without multivariable adjustment, leaving room for residual confounding from unmeasured factors such as socioeconomic status, tumor burden, or adjuvant therapy timing. Multiple testing without correction may elevate the risk of type I error. Blinding of participants and staff was not feasible given the intervention’s nature, potentially introducing expectancy effects. Furthermore, follow-up was restricted to the early postoperative phase. Considering that functional and psychological recovery in bone tumor patients often extends over many months or even years, the lack of medium- and long-term data (e.g., 1-year and 2-year assessments) limits the ability to judge the sustained effects of CNC. Future prospective studies with extended follow-up are therefore warranted to validate the durability of these benefits.

## 5. Conclusions

Comprehensive nursing care significantly enhanced postoperative functional recovery, daily living independence, and psychological well-being in patients undergoing bone tumor surgery. Compared with standard care, it yielded greater improvements across all functional and emotional domains, underscoring its value as an integrated intervention to optimize early rehabilitation and support holistic recovery in this population. However, as follow-up in this study was limited to the early postoperative phase, the long-term sustainability of these benefits remains uncertain, and future prospective studies with extended follow-up are warranted.

## Author contributions

**Conceptualization:** Xin Xu, Xin Wang, Quanhui Liu, Lu Xie.

**Data curation:** Xin Xu, Xin Wang, Quanhui Liu, Lu Xie.

**Formal analysis:** Xin Xu, Xin Wang.

**Funding acquisition:** Quanhui Liu.

**Investigation:** Xin Xu, Quanhui Liu, Lu Xie.

**Methodology:** Xin Xu, Xin Wang, Quanhui Liu, Lu Xie.

**Software:** Lu Xie.

**Supervision:** Xin Wang, Quanhui Liu, Lu Xie.

**Validation:** Xin Wang, Quanhui Liu.

**Visualization:** Xin Xu, Xin Wang, Quanhui Liu, Lu Xie.

**Writing – original draft:** Xin Xu, Lu Xie.

**Writing – review & editing:** Xin Xu, Lu Xie.

## References

[1] Qi L Ren X Liu Z. Predictors and survival of patients with osteosarcoma after limb salvage versus amputation: a population-based analysis with propensity score matching. World J Surg. 2020;44:2201–10. 32170370 10.1007/s00268-020-05471-9

[2] Evans DR Lazarides AL Visgauss JD. Limb salvage versus amputation in patients with osteosarcoma of the extremities: an update in the modern era using the national cancer database. BMC Cancer. 2020;20:995. 33054722 10.1186/s12885-020-07502-z PMC7557006

[3] Huayllani MT Restrepo DJ Boczar D. What factors define limb salvage or amputation surgery in osteosarcoma of the upper extremities? Anticancer Res. 2019;39:6807–11. 31810946 10.21873/anticanres.13896

[4] Traven SA Brinton DL Walton ZJ Leddy LR. A propensity-score matched analysis of limb salvage vs amputation for osteosarcoma. J Surg Oncol. 2019;120:1252–8. 31486107 10.1002/jso.25701

[5] Li Z Xu B Cai J Zha Z. Cancer-specific survival after limb salvage versus amputation in children and adolescents with osteosarcoma: a population-based analysis with propensity score matching. J Oncol. 2023;2023:8635829. 37089259 10.1155/2023/8635829 PMC10118882

[6] Wang L Fan Y Zhou Y Zhong G. Prognosis of limb‑salvage treatment of osteosarcoma in adolescent patients: a meta‑analysis. Oncol Lett. 2023;26:466. 37780543 10.3892/ol.2023.14053 PMC10534281

[7] Penrod D Hirsch B. Nursing care for metastatic bone cancer: trends for the future. Int J Environ Res Public Health. 2023;20:6483. 37569024 10.3390/ijerph20156483 PMC10418383

[8] Andritsch E Beishon M Bielack S. ECCO essential requirements for quality cancer care: soft tissue sarcoma in adults and bone sarcoma. a critical review. Crit Rev Oncol Hematol. 2017;110:94–105. 28109409 10.1016/j.critrevonc.2016.12.002

[9] Golemac M Yilmaz M Petersen MM. Postoperative challenges addressed through nursing care of patients receiving lower extremity tumor prosthesis. BMC Nurs. 2024;23:714. 39367361 10.1186/s12912-024-02400-2 PMC11452984

[10] Yu TY Wu TJ Jou ST Lee CY Sheih CS Chen CW. Examining the emotional healing process through bibliotherapy in adolescents with cancer: a qualitative descriptive study. Eur J Oncol Nurs. 2024;71:102653. 38991357 10.1016/j.ejon.2024.102653

[11] Roberts N Lee V Ananng B Körner A. Acceptability of bibliotherapy for patients with cancer: a qualitative, descriptive study. Oncol Nurs Forum. 2016;43:588–94. 27541551 10.1188/16.ONF.588-594

[12] Haynes KK Rosenthal HG. The ever-changing world of limb salvage surgery for malignant bone tumors. Nurs Clin North Am. 2020;55:251–66. 32389258 10.1016/j.cnur.2020.02.006

[13] Yang P Su Y Zhao W. Pain management of newly diagnosed sarcoma patients at a single center. Medicine (Baltim). 2022;101:e31422. 10.1097/MD.0000000000031422 PMC9750675 36626440

[14] Sun H Li Y Liu K. Effect of perioperative nursing for artificial knee replacement on patients with osteosarcoma of the distal femur. Am J Transl Res. 2021;13:10356–62. 34650703 PMC8507082

[15] Dong L Sun Y Hu J. Evidence-based nursing reduces complications and negative emotions and improves limb function in patients undergoing hip arthroplasty. Am J Transl Res. 2023;15:1779–88. 37056799 PMC10086896

[16] Liu X Sun L Du X Zhang C Zhang Y Xu X. Reducing anxiety and improving self-acceptance in children and adolescents with osteosarcoma through group drawing art therapy. Front Psychol. 2023;14:1166419. 37139009 10.3389/fpsyg.2023.1166419 PMC10149726

[17] Liu H Gao X Hou Y. Effects of mindfulness-based stress reduction combined with music therapy on pain, anxiety, and sleep quality in patients with osteosarcoma. Braz J Psychiatry. 2019;41:540–5. 31116262 10.1590/1516-4446-2018-0346 PMC6899366

[18] Wu TJ Sheih CS Jou ST. Effects of bibliotherapy on emotional distress, coping strategies, and resilience in adolescents with cancer: A pilot randomized controlled trial. Eur J Oncol Nurs. 2025;76:102900. 40381380 10.1016/j.ejon.2025.102900

